# Chromatographic Fingerprint Analysis and Effects of the Medicinal Plant Species *Mitracarpus frigidus* on Adult *Schistosoma mansoni* Worms

**DOI:** 10.1155/2014/941318

**Published:** 2014-05-08

**Authors:** Rodrigo Luiz Fabri, Danielle Maria de Oliveira Aragão, Jônatas Rodrigues Florêncio, Nícolas de Castro Campos Pinto, Ana Carolina Alves Mattos, Paulo Marcos Zech Coelho, Maria Christina Marques Nogueira Castañon, Eveline Gomes Vasconcelos, Priscila de Faria Pinto, Elita Scio

**Affiliations:** ^1^Bioactive Natural Products Laboratory, Department of Biochemistry, Institute of Biological Sciences, Federal University of Juiz de Fora, 36036 900 Juiz de Fora, MG, Brazil; ^2^Schistosomiasis Laboratory, René Rachou Research Center, FIOCRUZ, 30190 002 Belo Horizonte, MG, Brazil; ^3^Department of Morphology, Institute of Biological Sciences, Federal University of Juiz de Fora, 36036 900 Juiz de Fora, MG, Brazil; ^4^Protein Structure and Function Study Laboratory, Department of Biochemistry, Institute of Biological Sciences, Federal University of Juiz de Fora, 36036 900 Juiz de Fora, MG, Brazil

## Abstract

The aims of this work were to evaluate the *in vitro* and *in vivo* schistosomicidal properties of the methanolic extract of the aerial parts of *Mitracarpus frigidus* (MFM) and to determine its HPLC profile. For the *in vitro* experiment, four pairs of adult worms, obtained from infected mice, were exposed to different concentrations of MFM (100 to 400 **μ**g/mL) for 24 and 48 h and analyzed under an inverted microscope. For the *in vivo* experiment, mice were inoculated with cercariae and, 20 days after infection, MFM (100 and 300 mg/kg) was administered orally for the following 25 days. Mice were euthanized after 60 days. MFM showed *in vitro* schistosomicidal activity, exhibiting the opening of the gynaecophoral canal of some male schistosomes, the presence of contorted muscles, vesicles, and the darkening of the paired worms skin. *In vivo* experiments showed that MFM treatments significantly reduced total worm count, as praziquantel, showing a decrease in liver and spleen weight. Also, a significant reduction in granuloma density was observed. MFM treatment did not cause alterations in the liver function of either infected or noninfected mice. The HPLC chromatogram profile showed the presence of kaempferol-*O*-rutinoside, rutin, kaempferol, psychorubrin, and ursolic acid.

## 1. Introduction

Schistosomiasis, an infection caused by trematode worms of the genus* Schistosoma*, is considered one of the most significant neglected tropical diseases in the world [1]. It is estimated that 779 million people are at risk for schistosomiasis, with 230 million infected in 77 countries and territories [1–3].

The current treatment is based on the use of praziquantel and oxamniquine [4, 5]. Those drugs are effective against all species of schistosome; however, they do not prevent reinfection, are inactive against juvenile schistosomes, and have only a limited effect on the already developed liver and spleen lesions [6–8]. Praziquantel has a key role in population based disease control programs in most endemic countries [3, 9, 10]. The* in vitro* mechanism of action of this drug on adult* S. mansoni* worms has been well-described in the literature. This drug can cause muscle contraction and promote the immediate death of adult worms, miracidia, and primary sporocysts [11, 12]. However, the worryingly small portfolio of treatment options and the inevitability of resistance now that mass-administration programmes are in effect [13, 14] and the hemorrhage caused by this drug in the host lung tissue, as well as abdominal pain and diarrhea [15, 16], reinforce the need to develop new, safe, and effective schistosomicidal drugs. In this regard, the search for bioactive natural products against the schistosome has been intensified to establish future strategies to control schistosomiasis [17–20].

*Mitracarpus frigidus* (Willd. ex Roem. & Schult.) K. Shum is a species of the family Rubiaceae found throughout South America and, in Brazil, this species can be found in all states [21]. The methanolic extract of aerial parts (MFM) revealed the presence of flavonoids, tannins, alkaloids, terpenes, and quinones and showed antimicrobial, leishmanicidal, cytotoxic, and laxative activities. Moreover, MFM revealed no toxicity signs in rat models [22, 23]. Recently, the pyranonaphthoquinone psychorubrin was firstly isolated from this species [24].

However, there is no scientific report available in the literature on the anti-*Schistosoma mansoni* activity of* M. frigidus*. In view of this, the present study aimed to investigate the* in vitro* and* in vivo* shistosomicidal activity of the* M. frigidus* methanolic extract (MFM) obtained from the aerial parts in* Schistosoma mansoni*-infected mice. Furthermore, hematological, biochemical, and parasitological parameters were also determined.

## 2. Materials and Methods

### 2.1. Plant Material and Extraction

*Mitracarpus frigidus *aerial parts, collected in Juiz de Fora, Minas Gerais, Brazil, in May 2011, were identified by Dr. Tatiana Konno from the Nucleus of Ecology and Socio-Environmental Development of Macaé, Federal University of Rio de Janeiro. A voucher specimen (CESJ 46076) was deposited at the Herbarium Leopoldo Krieger of the Federal University of Juiz de Fora. Oven-dried and powdered aerial parts of the plant (1000 g) were extracted by maceration with methanol (5 × 2000 mL) for five days at room temperature and the methanolic extract (MFM) was obtained by evaporation (yield 10% w/w).

### 2.2. *In Vitro* Studies with* Schistosoma mansoni*

Swiss mice were individually infected subcutaneously with 100 cercariae/animal of the LE strain of* Schistosoma mansoni* (FIOCRUZ, Belo Horizonte, Brazil) in order to obtain the adult worms. The type of infection realized was bisexual, resulting in adult male and female worms. After 50 days, the infected animals were euthanized using a solution of 3% sodium pentobarbital (30 *μ*L/animal), and the worms were obtained by hepatic portal system perfusion according to the technique described by Smithers and Terry [25]. The Ethical Committee of the Federal University of Juiz de Fora, Juiz de Fora, MG, Brazil, protocol number 017/2009, approved these studies.

#### 2.2.1. Viability Assay

All the procedures conducted after worm extraction were made under aseptic conditions, including equipment and solutions. The worms were washed in RPMI-1640 medium to remove the perfusion detritus. After washing, four live worm pairs showing intense motility were transferred to each well of a 24-well culture plate containing 4 mL of RPMI-1640 medium supplemented with 5% of fetal calf serum and 100 *μ*g/mL of penicillin/streptomycin [26]. The pairs were exposed to increasing concentrations of MFM (100, 200, and 400 *μ*g/mL), and the worms were kept in contact with the extracts for 24 h. In the first experiment, the analyses were performed 6 and 24 h after the addition of 200 or 400 *μ*g/mL of MFM and 24 h after removal of the extracts. In the second experiment, the analyses were conducted 24 h after the addition of 100 or 200 *μ*g/mL of MFM and 24 and 48 h after their removal. After removal of the extracts, the worms were washed three times with the culture medium and then maintained in culture. In both experiments, the negative control group was included, comprising four pairs of worms in each well, in the presence of 1% DMSO (v/v) in 0.9% NaCl solution. During the entire assay, the worms were maintained in an incubator at 37°C and an atmosphere containing 5% of CO_2_. Four independent experiments were performed. Analyses were carried out using an inverted Olympus microscope and photographed with digital camera Canon.

### 2.3. *In Vivo* Schistosomicidal Analyses

#### 2.3.1. Experimental Design

Eighty female Swiss mice, weighing between 20 and 30 g, were used in this experiment. The mice were divided into eight groups (*n* = 10): four groups treated and noninfected and four groups treated and infected. The animal groups were infected with approximately 50 cercariae/animal (LE/BH strain), as described by Araújo et al. [10]. To evaluate the possible toxicity of the different treatments, noninfected animals were divided into (A) negative control group treated with a 1% DMSO (v/v) in 0.9% NaCl solution, (B) positive control group treated with praziquantel (200 mg/kg), and (C) and (D) groups treated with MFM at 100 and 300 mg/kg diluted with saline + 1% DMSO, respectively. The infected animals were divided into (E) negative control group treated with a DMSO 1% (v/v) in NaCl 0.9% solution, (F) positive control group treated with praziquantel (200 mg/kg), and (G) and (H) groups treated with MFM at 100 and 300 mg/kg diluted with saline + 1% DMSO, respectively. All of the treatments started on the twentieth day after infection. MFM and the negative control were administered in one daily dose for 25 days. The positive control, praziquantel, was administered in a single dose. At the end of the treatment period, the animals were maintained for a period of 15 days, completing 60 days of infection.

#### 2.3.2. Determination of Parasite Load and Biochemical and Hematological Parameters

After 60 days of cercarial exposure, all animals were weighed and anesthetized and blood samples were collected for assessment of biochemical and hematological parameters. Immediately after this procedure, the animals were euthanized, and the adult worms recovered from the portal and mesenteric veins by perfusion. In addition, the liver and spleen were removed and weighed. The measurement of biochemical parameters was performed using commercial kits (BIOCLIN and LABTEST) and included aspartate aminotransferase (AST), alanine aminotransferase (ALT), alkaline phosphatase (ALP), total protein, albumin, and globulin. Hematological parameters (total and specific leukocytes count) were also performed.

#### 2.3.3. Histological Analysis

Transverse sections of all liver lobes of infected mice (*n* = 5 per group) were collected, fixed in 4% buffered formaldehyde solution, and embedded in paraffin. Sections of 5–10 *μ*m were stained with haematoxylin and eosin (H&E). For the evaluation of granuloma density, stained slides were observed using bright field microscopy and all granulomas containing central viable eggs were quantified. All evaluations were blind performed by two different observers [27]. The area of hepatic granuloma was determined in histological sections from 20 to 30 granulomas per animal, containing central viable eggs, randomly chosen. The granuloma area was manually delimited, captured by a CCD camera using bright field microscopy, and automatically processed with IMAGE-PROPLUS.

### 2.4. High Pressure Liquid Chromatography (HPLC) Analysis

HPLC analysis was performed using an Agilent Technologies 1200 Series, with a PDA detector and an automatic injector. The column employed was a Zorbax SB-18; 250 × 4.6 mm, 5 *μ*m particle size. Solvents that constituted the mobile phase were A (water pH adjusted to 4.0 with H_3_PO_4_) and B (acetonitrile). The elution conditions applied were 0–20 min, 5–80% B and 20–30 min, 80–95% B. The mobile phase was returned to the original composition over the course of 30 min, and an additional 5 min was allowed for the column to reequilibrate before injection of the next sample. The sample volume was 20 *μ*L at a concentration of 1 mg/mL and a flow rate of 1 mL/min and the temperature was maintained at 25°C during the analysis. Detection was performed simultaneously at 210, 230, 254, and 280 nm. Four pure standards kaempferol, kaempferol-*O*-rutinoside, rutin, and ursolic acid, previously identified in* Mitracarpus *genus [28, 29], were used in this experiment as markers, and psychorubrin which was isolated from this species was also added [24]. For all experiments, MFM and the standards were dissolved in methanol.

### 2.5. Statistical Analysis

Values are presented as means ± SEM. Statistical differences between the treatments and the controls were tested by one-way analysis of variance (ANOVA), followed by the Bonferroni test using the “GraphPad Prism 4” statistic computer program. A difference in the mean values of *P* < 0.05 was considered to be statistically significant.

## 3. Results and Discussion

### 3.1. *In Vitro* Studies with* Schistosoma mansoni*

The profile of the damage caused by the exposure of the adult worms of* S. mansoni* to medicinal plants extracts can be determined through the observation of reduced motility, incapacity of adhesion in the culture plate by sucker cup, and tegument darkening [10, 12, 30, 31]. When the motility is lost, the worms can be considered dead [26].

**Figure 1:**
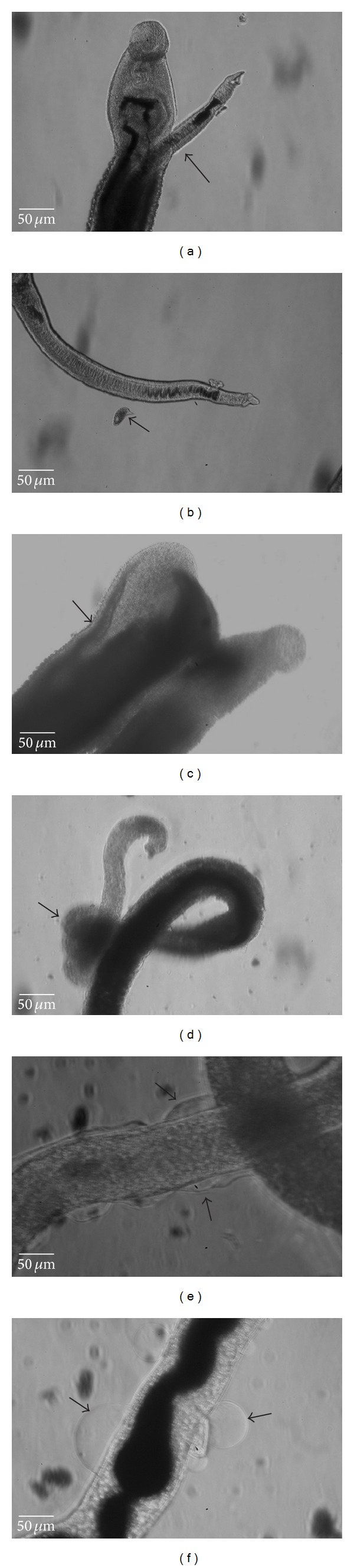
*In vitro* schistosomicidal activity of* Mitracarpus frigidus* methanolic extract (MFM) at 100 *μ*g/mL concentration after 24 hours of incubation. (a) Paired worms incubated in culture medium—the arrow shows pairs united in gynecophoral canal; (b) female worm incubated only with culture medium, showing eggs in the first stage of growth (arrow); (c) male worm showing fully open gynecophoral canal (arrow); (d) female worm showing contorted muscles; (e) and (f) presence of vesicles in female worm tegument sections.

The morphological characteristics of the paired worms of* S. mansoni* maintained in culture medium with 1% DMSO (v/v) in 0.9% NaCl solution (negative control group) and in the presence of MFM (100 *μ*g/mL) are shown in Figure 1. After 48 h exposure, the pairs of worms in the control group (Figures 1(a) and 1(b)) continued mating and showing active movements, without lesions in the tegument, with the presence of eggs in the culture medium. On the other hand, after 24 h of exposure to MFM at concentrations of 100, 200, and 400 *μ*g/mL, the worms showed complete paralysis, including the loss of movement of the suction cups with darkening in the tegument and the death of all parasites. Figures 1(c), 1(d), 1(e), and 1(f) show the morphological changes occurring after exposure of pairs of adult worms to MFM at 100 *μ*g/mL. These changes included the opening of the gynecophoral canal of some males (Figure 1(c)), the presence of males and females with contorted muscles, the darkening of the skin (Figure 1(d)), and the presence of vesicles in some skin formation (Figures 1(e) and 1(f)). This type of damage was also observed at the concentrations of 200 and 400 *μ*g/mL of MFM (data not shown).

Therefore, due to the promising activity observed for MFM against adult worms for the* in vitro* experiments,* in vivo* studies were performed to observe its therapeutic potential and toxicity.

### 3.2. *In Vivo* Schistosomicidal Activity

In order to investigate the effect of MFM treatment on body weight gain of both normal and* S. mansoni-*infected mice, body weight was measured after 60 days of treatment. No significant difference was observed between infected and normal mice (Table 1).* S. mansoni* infection is caused by cercariae penetration in the human skin and the symptoms are due to eggs that migrate to the liver, being secreted by worms living in the mesenteric and portal veins which leads to hepatosplenomegaly [19, 32–34]. Therefore, in order to examine the effect of MFM on hepatosplenomegaly, the liver and spleen were excised from dissected mice after perfusion and weighed and the relative weight percentage was calculated. As shown in Table 1, there was no significant difference in the liver and spleen relative weights among the noninfected mice groups. Although the infected mice presented an increase in liver and spleen weights, the groups treated with MFM (Groups G and H) showed a significant decrease in both liver and spleen relative weights when compared to the respective negative control (Group E). Those results indicated that MFM treatment reduced the increase in the organs weights induced by* S. mansoni *infection.

**Figure 2:**
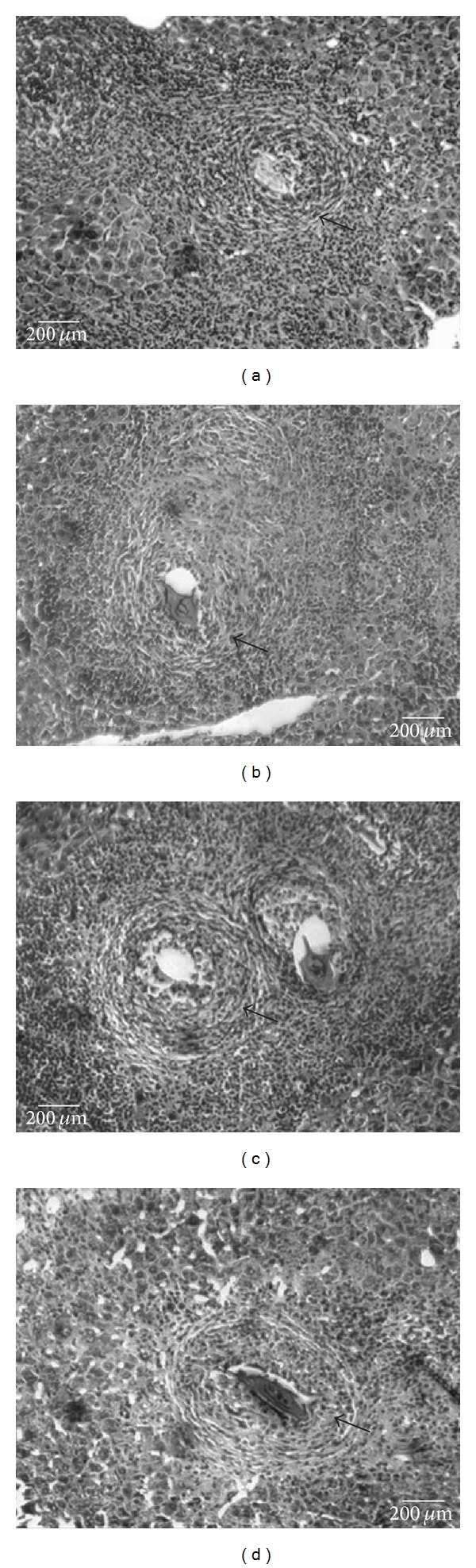
Effects of* Mitracarpus frigidus* methanolic extract on hepatic granuloma. At 60 days of infection, the hepatic tissues were collected and used for morphological study of the granulomatous area. All granulomas containing a central viable egg were measured and photographed. In (a) general aspects of the hepatic granulomas obtained from infected and untreated animals; (b) the infiltrate around the granuloma in treated animals with a single dose (200 mg/kg) of praziquantel is shown. In ((c), 100 mg/Kg) and ((d), 300 mg/kg) the granulomas from infected and treated animals after 20 days with different doses of the* M. frigidus* extract, showing that there are no changes in their structure and granulomatous infiltrate.

In addition, a significant reduction in granuloma density was observed in the groups treated with MFM (Groups G and H) when compared with the respective negative control (Group E). These results were comparable to that found for the mice treated with praziquantel (Table 1 and Figure 2).

Histological examination of the H&E stained liver sections showed that the granulomas of the infected negative control group (Group E) were composed of central ova surrounded by inflammatory cells associated with laminated layers of fibrous tissue at the periphery. In addition, severe necrosis was observed in the hepatic tissue (Figure 2(a)). On the other hand, the granulomas of the infected, treated mice (Groups F, G, and H) were observed as a concentric focus of mononuclear and polymorphonuclear cells around the egg, and the laminated layers of fibrous connective tissue nearly disappeared. Minimal microvascular changes and no hepatocyte necrosis were observed in the liver sections of those mice (Figures 2(b), 2(c), and 2(d)).

In order to evaluate the* in vivo* schistosomicidal effect of MFM, 60 days after cercarial exposure, the adult worms of* S. mansoni*-infected mice were recovered from the portal and mesenteric veins by perfusion and counted. As shown in Table 2, MFM treatments (100 and 300 mg/kg) significantly reduced total worm count (69 and 58%, resp.), as well as the reference group treated with praziquantel (49%) when compared with the control group. MFM reduced worm liver and mesentery burden to the extent of 91 and 65% at 100 mg/kg and by 65 and 58% at 300 mg/kg, respectively. By the other side, praziquantel reduced the liver and mesentery worm burden in 48% and 51%, respectively.

There are relatively few reports in the literature that show* in vivo* schistosomicidal activity of plant extracts [35, 36]. For example, artemether, an artemisinin derivative, used as a prophylactic agent against schistosomiasis japonica in China, at a concentration of 400 mg/kg, was able to reduce 60% of total worms during six days of treatment, in an experimental model [34]. El-Shenawy et al. [36] demonstrated that an alcoholic extract of* Cleome droserifolia *(Forssk.) Del. branches reduced 33% of worm burden at a concentration of 310 mg/kg. On the other hand, the ethanolic extract of* Nigella sativa *L., described in folk medicine as possessing hepatoprotective and antiprotozoal properties, was not able to reduce the number of worms after experimental infection [37].

In order to evaluate the ameliorative effect of MFM treatment on liver pathology induced by* S. mansoni* infection, the levels of total protein content and ALT, AST, and ALP activity were measured in the serum. Total protein content of noninfected mice treated with MFM and praziquantel (Groups B, C, and D) was comparable to the respective control group (Group A) (Table 3). On the other hand, the treated and infected mice (Groups F, G, and H) presented a protein content much lower than the noninfected groups. A decrease in total serum protein in the infected animals is attributed to the liver damage caused by infection [36, 38].

The level of globulin increased significantly in the infected group treated with MFM at 100 mg/kg (Group G) compared to the negative control (Group E) and the praziquantel treated (Group F) groups. This increase in globulin level may represent a responsive mechanism enhancing the immunity of the host [39]. However, there was no significant difference in albumin levels among the infected groups. The A/G ratio content for the infected mice treated with MFM at 100 mg/kg (Group G) is comparable to the negative control group (Group E) and lower than the praziquantel treated group (Table 3).

AST, ALT, and ALP levels in the infected treated mice (Groups F, G, and H) were significantly lower than those of the respective control group (Group E). These observations could be attributed to the reduction in hepatic granuloma and fibrosis, as well as the absence of necrotic liver tissue, in the infected treated mice. For noninfected mice, these enzymes remained at normal levels (Table 3 and Figure 2). The results indicated that the administration of MFM did not cause alterations in the liver function of either the infected or the noninfected mice.

Evaluation of cell profile during infection is one of the strategies to assess which cells are stimulated by events which modify the inflammatory process. The mechanism of selective recruitment of leukocytes to the inflamed tissue is related to chemotactic factors [40]. As depicted in Table 3, there was a significant decrease in total leukocyte count in the infected treated mice (Groups F, G, and H) when compared to the respective control group (Group E). Although MFM was not able to reduce the granulomatous area, it reduced the number of liver worms and, consequently, the recruitment of leukocytes.

The specific leukometry showed that MFM did not significantly affect the basophil, neutrophil, lymphocyte, or eosinophil count in the noninfected, treated mice (Groups B, C, and D), compared to the respective control (Group A). The neutrophil counts of the infected, treated mice (Groups F, G, and H) were significantly increased, but the eosinophil and monocyte counts were significantly decreased, compared with the negative control (Group E). Therefore, eosinophil and monocyte counts are usually increased in helminthic diseases; those results are in agreement with the reduction of the worm burden caused by MFM.

### 3.3. HPLC Fingerprint

**Figure 3:**
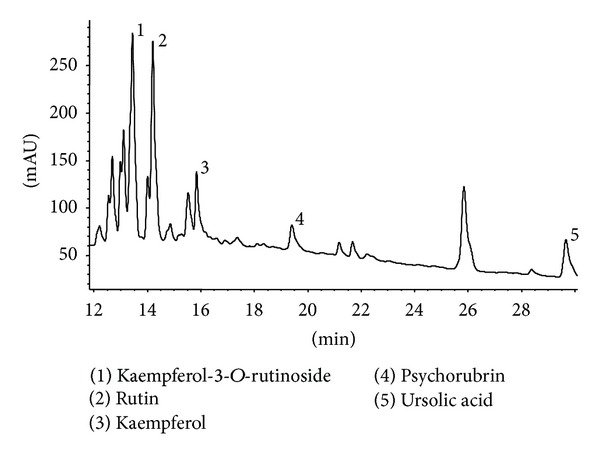
HPLC chromatogram of* Mitracarpus frigidus* methanolic extract (MFM). The analysis was performed using a linear gradient of a binary solvent system A (water pH adjusted to 4.0 with H_3_PO_4_) : B (acetonitrile). The elution conditions applied were 0–20 min, 5–80% B and 20–30 min, 80–95% B. It was run at a flow rate of 1 mL/min over 30 minutes, with an injection volume (“loop”) of 20 *μ*L and UV detection was at 230 nm.

Under the experimental conditions, the HPLC chromatogram determined for MFM is shown in Figure 3. Five peaks were detected as kaempferol-*O*-rutinoside, rutin, kaempferol, psychorubrin, and ursolic acid.

This result strongly suggested that kaempferol contributed to MFM schistosomicidal activity. Braguine et al. [41] showed that this compound is able to separate coupled* S. mansoni* adult worms and to kill adult schistosomes* in vitro* at 100 *μ*M. Also, there are reports on the antihelminthic activity of ursolic acid [42], but, according to Alvarenga et al. [43], this compound is not active against* S. mansoni *adult worms. However, it is noteworthy to mention the well-documented hepatoprotective properties of ursolic acid, due to the enhancement of the body defense systems [44], which might have contributed to the lack of alterations in liver function of infected and noninfected mice treated with MFM. In addition, the potent antioxidant effects of rutin [45], which may be helpful against the oxidative liver tissue damage often caused by* S. mansoni* infection [46], are well known.

## 4. Conclusions

These results demonstrated that* Mitracarpus frigidus* might be interesting in schistosomiasis treatment, as it decreased considerably the disease severity by reducing significantly the parasite load without altering liver function. Further studies designed to isolate, identify, and characterize the active constituents of MFM may provide a better understanding of its schistosomicidal mechanism.

## Figures and Tables

**Table 1:** Effects of *Mitracarpus frigidus* methanolic (MFM) extract treatment on body weight, relative organ weights, and granuloma formation, after 60 days of infection.

	Non-infected groups				Infected groups			
	Group A	Group B	Group C	Group D	Group E	Group F	Group G	Group H
	Negative control	Praziquantel	MFM	MFM	Negative control	Praziquantel	MFM	MFM
		200 mg/kg	100 mg/kg	300 mg/kg		200 mg/kg	100 mg/kg	300 mg/kg
Body weight (g)	26.5 ± 0.5	24.5 ± 0.7	24.8 ± 0.9	25.3 ± 0.6	28.0 ± 0.5	27.9 ± 0.5	26.5 ± 0.5	24.5 ± 0.7
Relative liver weights	4.9 ± 0.2	5.4 ± 0.2	5.4 ± 0.1	5.4 ± 0.2	12.8 ± 0.7	12.0 ± 0.4	4.9 ± 0.2	5.4 ± 0.2
Relative spleen weights	0.3 ± 0.04	0.3 ± 0.04	0.4 ± 0.03	0.5 ± 0.09	3.4 ± 0.2	2.8 ± 0.3	0.3 ± 0.04	0.3 ± 0.04
Number of granulomas	—	—	—	—	62.2 ± 1.6	45.1 ± 3.1^a^	—	—
Mean granuloma diameter (*μ*m)	—	—	—	—	7.4 ± 0.3	8.8 ± 0.1^a^	—	—

The values shown are mean ± SEM (*n* = 10). ^a^Statistically different from the infected, negative control group (E) (ANOVA followed by Bonferroni, *P* < 0.05).

**Table 2:** Results obtained in mice experimentally infected with 50 ± 10 cercariae of *Schistosoma mansoni *(LE strain) treated with *Mitracarpus frigidus*, orally, after 60 days of infection.

Groups	Worm distribution					
	Liver		Mesentery		Total	
	Means of worms (M/F)	Reduction^c^ (%)	Means of worms (M/F)	Reduction^c^ (%)	Global means (M/F)	Global reduction^c^ (%)
Negative control	4.6 ± 1.0	—	19.2 ± 1.4	—	23.0 ± 1.0	—
Praziquantel 200 mg/kg	2.4 ± 0.7^a^	48	9.4 ± 1.7^a^	51	11.8 ± 2.0^a^	49
MFM 100 mg/kg	0.4 ± 0.4^a,b^	91	6.8 ± 1.6^a^	65	7.2 ± 1.9^a^	69
MFM 300 mg/kg	1.6 ± 0.8^a^	65	8.0 ± 1.5^a^	58	9.6 ± 1.7^a^	58

The values shown are mean ± SEM (*n* = 8). ^a^Statistically different from the negative control group. ^b^Statistically different from the positive control group (praziquantel) (ANOVA followed by the Bonferroni test, *P* < 0.05). ^c^Percentage reduction (%) = {1 − (mean of worms in the negative control group/mean of worms in the groups treated)}  × 100.

**Table 3:** Effects of *Mitracarpus frigidus* methanolic extract (MFM) treatment on the biochemical and hematological parameters, after 60 days of infection.

	Noninfected groups				Infected groups			
	Group A	Group B	Group C	Group D	Group E	Group F	Group G	Group H
	Negative control	Praziquantel	MFM	MFM	Negative control	Praziquantel	MFM	MFM
		200 mg/kg	100 mg/kg	300 mg/kg		200 mg/kg	100 mg/kg	300 mg/kg
Total protein (g/dL)	13.2 ± 0.2	10.6 ± 0.3	12.5 ± 0.5	11.4 ± 0.5	5.1 ± 0.2	4.8 ± 0.2	5.7 ± 0.2	5.4 ± 0.2
Albumin (g/dL)	4.4 ± 0.4	3.0 ± 0.2^a^	2.3 ± 0.1^a,b^	1.8 ± 0.1^a,b^	2.8 ± 0.2	2.7 ± 0.2	2.7 ± 0.2	3.1 ± 0.2
Globulin (g/dL)	7.9 ± 1.4	7.6 ± 0.5	10.2 ± 0.6^a,b^	9.8 ± 0.4^a,b^	2.3 ± 0.2	2.0 ± 0.3	3.0 ± 0.3^c,d^	2.4 ± 0.3
A/G	0.5 ± 0.1	0.4 ± 0.05	0.2 ± 0.02	0.2 ± 0.02	1.3 ± 0.3	2.1 ± 0.6^c^	1.0 ± 0.1^d^	1.6 ± 0.3
ALP (U/L)	17.5 ± 1.5	22.7 ± 2.1	12.2 ± 1.4^b^	7.7 ± 1.9^a,b^	55.1 ± 5.0	38.7 ± 3.2^c^	38.7 ± 3.9^c^	41.7 ± 3.4^c^
AST (U/L)	10.9 ± 1.3	18.6 ± 4.3^a^	8.5 ± 1.4^b^	13.2 ± 3.1	44.6 ± 3.8	21.0 ± 3.5^c^	26.8 ± 1.9^c^	32.3 ± 3.0^c,d^
ALT (U/L)	17.9 ± 2.8	9.3 ± 1.6^a^	11.6 ± 2.7	6.3 ± 1.0^a^	33.3 ± 2.3	29.8 ± 3.1	4.2 ± 0.4^c,d^	14.6 ± 3.5^c,d^
Total leukocytes (10^3^/*μ*L)	5.1 ± 0.4	4.3 ± 0.4	4.8 ± 0.4	5.0 ± 0.4	8.4 ± 0.6	5.6 ± 0.7^c^	5.7 ± 0.4^c^	5.2 ± 0.4^c^
Basophil (%)	1.0 ± 0	1.0 ± 0	1.0 ± 0	1.0 ± 0	1.0 ± 0	1.1 ± 0.1	1.2 ± 0.1	1.2 ± 0.1
Eosinophil (%)	2.1 ± 0.4	2.3 ± 0.2	1.9 ± 0.1	2.1 ± 0.1	20.4 ± 1.2	11.2 ± 1.3^c^	5.1 ± 1.0^c,d^	6.5 ± 1.0^c,d^
Monocyte (%)	9.4 ± 0.5	7.4 ± 0.7	5.4 ± 0.7^a^	5.2 ± 1.2^a^	19.6 ± 2.2	10.3 ± 1.3^c^	4.6 ± 1.0^c,d^	9.1 ± 1.4^c^
Neutrophil (%)	43.3 ± 3.5	53.6 ± 2.5^a^	49.0 ± 1.4	49.7 ± 2.3	39.8 ± 2.6	59.4 ± 2.3^c^	65.8 ± 2.0^c^	65.4 ± 1.4^c^
Lymphocyte (%)	42.4 ± 2.6	34.8 ± 2.5^a^	45.8 ± 2.7^b^	47.2 ± 2.0^b^	17.4 ± 2.5	13.9 ± 1.2	20.5 ± 1.5^d^	16.9 ± 1.3

The values shown are mean ± SEM (*n* = 10). ^a^Statistically different from the noninfected, negative control Group A. ^b^Statistically different from the noninfected, positive control Group B. ^c^Statistically different from the infected, negative control Group E. ^d^Statistically different from the infected, positive control Group F (ANOVA followed by the Bonferroni test, *P* < 0.05).

## References

[1] Vimieiro AC, Araújo N, Katz N, Kusel JR, Coelho PM (2013). Schistogram changes after administration of antischistosomal drugs in mice at the early phase of *Schistosoma Mansoni* infection. *Memórias do Instituto Oswaldo Cruz*.

[2] Nascimento GL, de Oliveira MR (2014). Severe forms of schistosomiasis mansoni: epidemiologic and economic impact in Brazil, 2010. *Transactions of the Royal Society of Tropical Medicine and Hygiene*.

[3] World Health Organization Schistosomiasis.

[4] Cioli D, Pica-Mattoccia L, Archer S (1995). Antischistosomal drugs: past, present and future?. *Pharmacology and Therapeutics*.

[5] Cioli D, Botros SS, Wheatcroft-Francklow K (2004). Determination of ED50 values for praziquantel in praziquantel-resistant and -susceptible *Schistosoma Mansoni* isolates. *International Journal for Parasitology*.

[6] Fallon PG, Doenhoff MJ (1994). Drug-resistant schistosomiasis: resistance to praziquantel and oxamniquine induced in *Schistosoma Mansoni* in mice is drug specific. *The American Journal of Tropical Medicine and Hygiene*.

[7] Stelma FF, Talla I, Sow S (1995). Efficacy and side effects of praziquantel in an epidemic focus of *Schistosoma Mansoni*. *The American Journal of Tropical Medicine and Hygiene*.

[8] Ismail M, Botros S, Metwally A (1999). Resistance to praziquantel: direct evidence from *Schistosoma Mansoni* isolated from egyptian villagers. *The American Journal of Tropical Medicine and Hygiene*.

[9] Savioli L, Renganathan E, Montresor A, Davis A, Behbehani K (1997). Control of schistosomiasis—a global picture. *Parasitology Today*.

[10] Araújo N, De Mattos ACA, Sarvel AK, Coelho PMZ, Katz N (2008). Oxamniquine, praziquantel and lovastatin association in the experimental Schistosomiasis mansoni. *Memorias do Instituto Oswaldo Cruz*.

[11] Couto FFB, Coelho PMZ, Arajo N, Kusel JR, Katz N, Mattos ACA (2010). Use of fluorescent probes as a useful tool to identify resistant *Schistosoma Mansoni* isolates to praziquantel. *Parasitology*.

[12] Almeida LM, Farani PG, Tosta LA (2012). *In vitro* evaluation of the schistosomicidal potential of *Eremanthus erythropappus* (DC) McLeisch (Asteraceae) extracts. *Natural Product Research*.

[13] Penido ML, Coelho PM, Nelson DL (1999). Efficacy of a new schistosomicidal agent 2-[(methylpropyl)amino]-1-octanethiosulfuric acid against an oxamniquine resistant *Schistosoma Mansoni* isolate. *Memorias do Instituto Oswaldo Cruz*.

[14] Ribeiro-dos-Santos G, Verjovski-Almeida S, Leite LCC (2006). Schistosomiasis—a century searching for chemotherapeutic drugs. *Parasitology Research*.

[15] Flisser A, McLaren DJ (1989). Effect of Praziquantel treatment on lung-stage larvae of *Schistosoma Mansoniin vivo*. *Parasitology*.

[16] Kabatereine NB, Kemijumbi J, Ouma JH (2003). Efficacy and side effects of praziquantel treatment in a highly endemic *Schistosoma Mansoni* focus at Lake Albert, Uganda. *Transactions of the Royal Society of Tropical Medicine and Hygiene*.

[17] Ndamb J, Nyazema N, Makaza N, Anderson C, Kaondera KC (1994). Traditional herbal remedies used for the treatment of urinary schistosomiasis in Zimbabwe. *Journal of Ethnopharmacology*.

[18] Mølgaard P, Nielsen SB, Rasmussen DE, Drummond RB, Makaza N, Andreassen J (2001). Anthelmintic screening of Zimbabwean plants traditionally used against schistosomiasis. *Journal of Ethnopharmacology*.

[19] Sanderson L, Bartlett A, Whitfield PJ (2002). *In vitro* and *in vivo* studies on the bioactivity of a ginger (*Zingiber officinale*) extract towards adult schistosomes and their egg production. *Journal of Helminthology*.

[20] Mohamed AM, Metwally NM, Mahmoud SS (2005). Sativa seeds against *Schistosoma Mansoni* different stages. *Memorias do Instituto Oswaldo Cruz*.

[21] Pereira ZV, Carvalho-Okano RM, Garcia FCP (2006). Rubiaceae Juss. da Reserva Florestal Mata de Paraíso, Viçosa, MG, Brasil. *Acta Botanica Brasilica*.

[22] Fabri RL, Nogueira MS, Braga FG, Coimbra ES, Scio E (2009). Mitracarpus frigidus aerial parts exhibited potent antimicrobial, antileishmanial, and antioxidant effects. *Bioresource Technology*.

[23] Fabri RL, de Oliveira Aragão DM, Florêncio JR (2012). *In-vivo* laxative and toxicological evaluation and *in-vitro* antitumour effects of *Mitracarpus frigidus* aerial parts. *Journal of Pharmacy and Pharmacology*.

[24] Fabri RL, Grazul RM, Carvalho LO (2012). Antitumor, antibiotic and antileishmanial properties of the pyranonaphthoquinone psychorubrin from Mitracarpus frigidus. *Annals of the Brazilian Academy of Sciences*.

[25] Smithers SR, Terry RJ (1965). The infection of laboratory hosts with cercariae of *Schistosoma Mansoni* and the recovery of the adult worms. *Parasitology*.

[26] de Araújo SC, de Mattos ACA, Teixeira HF, Coelho PMZ, Nelson DL, de Oliveira MC (2007). Improvement of *in vitro* efficacy of a novel schistosomicidal drug by incorporation into nanoemulsions. *International Journal of Pharmaceutics*.

[27] Pyrrho AS, Lenzi HL, Ramos JA (2004). Dexamethasone treatment improves morphological and hematological parameters in chronic experimental schistosomiasis. *Parasitology Research*.

[28] Bisignano G, Sanogo R, Marino A (2000). Antimicrobial activity of *Mitracarpus scaber* extract and isolated constituents. *Letters in Applied Microbiology*.

[29] Gbaguidi F, Accrombessi G, Moudachirou M, Quetin-Leclercq J (2005). HPLC quantification of two isomeric triterpenic acids isolated from *Mitracarpus scaber* and antimicrobial activity on *Dermatophilus congolensis*. *Journal of Pharmaceutical and Biomedical Analysis*.

[30] Magalhães LG, Machado CB, Morais ER (2009). *In vitro* schistosomicidal activity of curcumin against *Schistosoma Mansoni* adult worms. *Parasitology Research*.

[31] Magalhães LG, Kapadia GJ, da Silva Tonuci LR (2010). *In vitro* schistosomicidal effects of some phloroglucinol derivatives from *Dryopteris* species against *Schistosoma Mansoni* adult worms. *Parasitology Research*.

[32] Jatsa HB, Sock ETN, Tchuente LAT, Kamtchouing P (2009). Evaluation of the *in vivo* activity of different concentrations of *Clerodendrum umbellatum* Poir against *Schistosoma Mansoni* infection in mice. *African Journal of Traditional, Complementary and Alternative Medicines*.

[33] Mata-Santos HA, Lino FG, Rocha CC, Paiva CN, Castelo Branco MTL, Pyrrho ADS (2010). Silymarin treatment reduces granuloma and hepatic fibrosis in experimental schistosomiasis. *Parasitology Research*.

[34] Abdul-Ghani R, Loutfy N, Sheta M, Hassan A (2011). Artemether shows promising female schistosomicidal and ovicidal effects on the Egyptian strain of *Schistosoma Mansoni* after maturity of infection. *Parasitology Research*.

[35] Hamed MA, Hetta MH (2005). Efficacy of *Citrus reticulata* and Mirazid in treatment of *Schistosoma Mansoni*. *Memorias do Instituto Oswaldo Cruz*.

[36] El-Shenawy NS, Soliman MFM, Abdel-Nabi IM (2006). Does *Cleome droserifolia* have anti-schistosomiasis mansoni activity?. *Revista do Instituto de Medicina Tropical de Sao Paulo*.

[37] El Shenawy NS, Soliman MFM, Reyad SI (2008). The effect of antioxidant properties of aqueous garlic extract and *Nigella sativa* as anti-schistosomiasis agents in mice. *Revista do Instituto de Medicina Tropical de Sao Paulo*.

[38] Guyton AC, Hall JE (2000). *Textbook of Medical Physiology*.

[39] El-Shenawy NS, Soliman MFM (2002). On the interaction between induced *Diabetes mellitus* and Schistosomiasis: mechanism and protection. *Egyptian Journal of Hospital Medicine*.

[40] Larocca RA, Souza BR, Marmol CHC (2004). Avaliação do recrutamento celular no modelo experimental da esquistossomose mansônica. *Ver UNIARA*.

[41] Braguine CG, Bertanha CS, Gonçalves UO (2012). Schistosomicidal evaluation of flavonoids from two species of *Styrax* against *Schistosoma Mansoni* adult worms. *Pharmaceutical Biology*.

[42] Zhou L, Wang J, Wang K (2012). Secondary metabolites with antinematodal activity from higher plants. *Studies in Natural Products Chemistry*.

[43] Alvarenga TA, Bêdo TRFO, Braguine CG (2012). Evaluation of *Cuspidaria pulchra* and its isolated compounds against *Schistosoma Mansoni* adult worms. *International Journal of Biotechnology for Wellness Industries*.

[44] Jie L (1995). Pharmacology of oleanolic acid and ursolic acid. *Journal of Ethnopharmacology*.

[45] Yang J, Guo J, Yuan J (2008). *In vitro* antioxidant properties of rutin. *LWT*.

[46] Ali HF (2007). Evaluation of antioxidant effect of *Citrus reticulata* in *Schistosoma Mansoni* infected mice. *Trends in Medical Research*.

